# Effect of Carbon on Void Nucleation in Iron

**DOI:** 10.3390/ma17133375

**Published:** 2024-07-08

**Authors:** Lin Shao

**Affiliations:** Accelerator Laboratory, Department of Nuclear Engineering, Texas A&M University, College Station, TX 77843, USA; lshao@tamu.edu

**Keywords:** void swelling, void nucleation, ion irradiation, neutron irradiation, impurity effect

## Abstract

The study reports the significance of carbon presence in affecting void nucleation in Fe. Without carbon, void nucleation rates decrease gradually at high temperatures but remain significantly high and almost saturated at low temperatures. With carbon present, even at 1 atomic parts per million, void nucleation rates show a low-temperature cutoff. With higher carbon levels, the nucleation temperature window becomes narrower, the maximum nucleation rate becomes lower, and the temperature of maximum void nucleation shifts to a higher temperature. Fundamentally, this is caused by the change in effective vacancy diffusivity due to the formation of carbon-vacancy complexes. The high sensitivity of void nucleation to carbon comes from the high sensitivity of void nucleation to the vacancy arrival rate in a void. The void nucleation is calculated by first obtaining the effective vacancy diffusivity considering the carbon effect, then calculating the defect concentration and defect flux change considering both carbon effects and pre-existing dislocations, and finally calculating the void nucleation rate based on the recently corrected homogeneous void nucleation theory. The study is important not only in the fundamental understanding of impurity effects in ion/neutron irradiation but also in alloy engineering for judiciously introducing impurities to increase swelling resistance, as well as in the development of simulation and modeling methodologies applicable to other metals.

## 1. Introduction

Subjected to ion, neutron, or electron irradiation, metals undergo the buildup of point defects in concentrations markedly surpassing their equilibrium levels. Consequently, small defect clusters nucleate and grow, evolving into complex defect structures like dislocations, dislocation loops, and voids. Understanding the response of metals to irradiation holds the utmost importance for both fission and fusion reactors, as irradiation causes material degradation [1]. Typical material degradation in fission reactors includes stress corrosion cracking [2], swelling [3], fuel restructuring [4], delayed hydride cracking [5], fracture [6], embrittlement [7], and creep [8]. Typical degradation expected in fusion reactors includes cracking, flaking, blistering, and embrittlement [9,10]. These phenomena greatly limit reactor performance and reactor lifetime. Concerning pressured water reactors and fast neutron reactors, the void swelling raises a substantial challenge regarding reactor safety. The process of swelling in irradiated metals typically includes three phases: an initial incubation period, a transient phase, and a phase of steady-state growth. While rich data exist for the steady-state growth phase, understanding of the first phase within the incubation period is very limited. Primarily, this is due to the challenge of identifying void embryos that fall beneath the detection threshold of conventional microscopy methods. Research concerning austenitic steels has revealed that the initial incubation phase of swelling is highly sensitive to slight variations in composition and minor deviations in thermal-mechanical treatments [11]. Furthermore, the duration of the incubation period is significantly influenced by environmental conditions, including the rate of displacement per atom (dpa), temperatures, and stresses. Gaining insights into void nucleation during the swelling incubation period is important for material development toward radiation-tolerant high-performance steels.

Among the numerous factors influencing void nucleation, the impact of impurities, especially carbon, holds significance. Recent studies indicate that a carbon concentration of ~100 atomic parts per million (appm) can alter the void distribution profile in irradiated iron [12]. Given that carbon is a common contaminant and is often utilized as an alloying element, whether intentionally or unintentionally, it becomes imperative to investigate its effects. The impact of carbon on interstitials is less pronounced when compared to its effect on vacancies [13,14,15]. Carbon can form bonds with a <110> Fe-Fe interstitial dumbbell, exhibiting a weak binding energy of approximately 0.5 eV [13]. Concerning vacancies, carbon exerts its influence through two possible mechanisms. First, carbon can elevate the migration energy of vacancies without trapping vacancies [16]. Second, carbon can bond with vacancies, resulting in the formation of C-V complexes [17]. The first mechanism influences the diffusivity of vacancies without altering their concentration. Conversely, the second mechanism doesn’t affect the diffusivity of free vacancies but reduces the number of mobile vacancies. Both effects can be represented by introducing effective vacancy diffusivity. The second effect is more significant and representative and can be modeled by considering various complex configurations beyond the interaction between a single vacancy and a single carbon atom.

The effect of carbon as a contaminant during prolonged accelerator ion irradiation has been a well-known issue. The problem is serious enough that accelerator laboratories involved in nuclear material testing have developed various methods to mitigate it. The accelerator laboratory at Los Alamos National Laboratory uses a coating technique [18], the accelerator laboratory at the University of Michigan employs plasma cleaning [19], and the accelerator laboratory at Texas A&M University uses multiple beam deflection [20]. Universal contamination may come from the beam line, target chamber, or the sample itself. Most likely, it is a combination of all, but the primary contamination source may vary for specific laboratories. Contamination on the sample surface, in one situation, can come from surface preparation, shipping, or pasting on the target holder.

The effect of carbon needs a thorough investigation. Additionally, there is a lack of a quantitative method to predict the level of severity under various carbon levels. Studying the effect of carbon on void swelling requires a multiscale modeling approach. The first step is to evaluate the effect of carbon on defect kinetics, which requires atomic-scale modeling. The second step is to use defect kinetics to predict void swelling. This step involves obtaining the defect population and defect flux under a given microstructure (i.e., pre-existing dislocation density).

Here, the sensitivity of void nucleation in α-iron (Fe) to carbon background levels is studied, driven by the need to evaluate the tolerance of impurity levels in both irradiation testing and material synthesis. Fe is selected for its representation of Fe-based steels. The former pertains to the credibility of accelerated irradiation testing for emulating neutron environments, while the latter pertains to the qualification of emerging manufacturing techniques such as additive manufacturing. 

## 2. Modeling Procedure

Void nucleation theory was previously established by Katz and Wiedersich [21] and Russel [22]. The mathematical treatment was recently revisited by Shao, and modification was proposed to fix a problem in the original treatment [23]. The original treatment uses a specific selection of energy reference points to simplify the expression. It, however, mistakenly removes an exponential modification function, leading to an underestimation of nucleation rates by orders of magnitude. Below, the original theory and recent corrections are explained as the foundation of the modeling procedure in the present work.

In homogenous void nucleation theory [21], the growth rate of voids from size x to the next size (x+1) for an arbitrary size function $f\left(x,t\right)$ considers the competing effects of four different interactions of voids with point defect, as described by the following:

(1) $$J\left(x,t\right)=\beta_{v}s\left(x\right)f\left(x,t\right)-\gamma_{v}\left(x+1\right)s\left(x+1\right)f(x+1,t)-\beta_{i}s\left(x+1\right)f\left(x+1,t\right)+\gamma_{i}\left(x\right)s\left(x\right)f(x,t)$$

where $J\left(x,t\right)$ is the void nucleation rate from size *x* to x+1, *x* is the number of vacancies contained in a void, *t* is time, $\beta_{v}$ and $\beta_{i}$ are the arrival rates of vacancies and interstitials to a void per unit area and unit time, respectively. $\gamma_{v}$ and $\gamma_{i}$ are the emission of vacancies and interstitials by a void per unit area and unit time. $s\left(x\right)$ is the surface area of a void of size x. Following the theoretical framework established by Katz, Wiedersich, and Russel [21,22], the void nucleation rate under a steady state growth is given by the following:

(2) $$J={\left[\sum_{x=1}^{x=\infty }\frac{1}{\beta_{v}s\left(x\right)n(x)}\right]}^{-1}$$

where n(x) is a specific constrained void size distribution satisfying J=0. Additional details of the derivation are provided in Appendix A. The J value from Equation (2) explicitly depends on vacancy concentration, as reflected by the appearance of $\beta_{v}$. Additionally, the value implicitly depends on interstitial concentrations, with their effect included in n(x). The function *n*(*x*) represents a specific size distribution of zero net growth flux. J=0 is achieved through the cancellation of flux contributions from both vacancy-driven and interstitial-driven growth [21]. $n\left(x\right)\;$has the solution, as follows:

(3) $$n\left(x\right)=n^{0}(x)\prod_{j=1}^{x-1}P_{j}$$

Additional details of the derivation are provided in Appendix A. The size distribution $n^{0}(x)$ represents another specific size function at which voids are in equilibrium with vacancies. The correction factor *P* is primarily determined by mathematical deviations, but it does possess a physical interpretation. *P* represents the ratio of the magnitude of the flow of void embryos when vacancies are in equilibrium with interstitials to the magnitude of the flow when actual interstitials are present. The magnitude of the flow is composed of the combined effects of vacancy-absorption-induced growth from size *x* to *x +* 1 and interstitial-absorption-induced reverse growth from size *x +* 1 to *x*, regardless of the flux direction. $n^{0}\left(x\right)$ is also called the constrained equilibrium distribution of voids [21]. It means the formation of a void size *x* from *x* vacancies does not change the total free energies. $n^{0}\left(x\right)$ was derived as the following [23]:

(4) $$n^{0}\left(x\right)=N\left[exp\frac{-W\left(x\right)+xW(1)}{kT}\right]{\left(\frac{n_{1}^{eq}}{N}\right)}^{x}{\left(S\right)}^{x}$$

where N is the atomic density of the system, k is the Boltzmann constant, T is temperature, and *S* is defined as vacancy supersaturation ratio (*S*$\equiv \frac{n_{1}}{n_{1}^{eq}}$). $n_{1}^{eq}$ is the equilibrium number of vacancies per unit volume. $W\left(x\right)$ is work needed to create a void of size x.

Equation (4) is different from the original formula in reference [21], which is expressed as follows:

(5) $$\;\;\;n^{0}\left(x\right)=Nexp[-\frac{W\left(x\right)-xkTlnS}{kT}]\;=Nexp[\frac{-W(x)}{kT}]S^{x}$$

The problem with Equation (5) in the original theory comes from the selection of a reference state. The theory was built upon the assumption that the system contains voids and vacancies reach equilibrium, in which there is no chemical potential change when *x* vacancies form a void of size *x*. In order to simplify the expression, Katz and Wiedersich set the chemical potential of a vacancy in the system containing $n_{1}^{eq}\;$as zero [21]. Consequently, the effect of chemical potential disappears. In contrast, the derivation shows chemical potentials change the base that is raised to the power of x in the expression of $n^{0}\left(x\right)$, introducing a size-dependent. In the correct equation, Equation (4), the effect of chemical potential is included in $n_{1}^{eq}$. More details of the derivation can be found in reference [23]. In a different way to explain, the original treatment ignores the effect of entropy in forming a vacancy, which is supposed to contribute a temperature-independent but size-dependent term in $n^{0}\left(x\right)$. This effect is included in ${\left(\frac{n_{1}^{eq}}{N}\right)}^{x}$ in Equation (4).

The energy required to form a void in pure Fe, $W\left(x\right),\;$can be obtained using various methods, including continuum mechanics models [21], first-principle quantum mechanics calculations [24], and molecular dynamics simulations (MD) [25]. The present study selected MD-obtained results for the work needed to create a void of size *x*, as given by the following [25]:

(6) $$W\left(x\right)=2.59x^{2/3}$$

The unit of $W\left(x\right)$ in Equation (6) is eV. The selection of MD-obtained results considers the fact that void swelling is frequently observed in pure Fe at temperatures around 500 °C [26]. MD-obtained kinetics yield a swelling temperature range in good agreement with experimental observations, as will be shown in this study.

Ab initio calculation suggests that the vacancy migration energy in body-center cubic (BCC) Fe is 0.73 eV [27]. If the vacancy migration energy is set at 0.73 eV and the vacancy formation energy at 2.59 eV [25], the activation energy for Fe self-diffusion in BCC Fe is about 3.32 eV. Note that this value matches the modeling result [27]. This value is also reasonably close to the experimental values ranging from 2.95 eV to 3.10 eV [28,29]. As a summary, Table 1 lists the parameters of pure Fe used in the present study. The pre-exponential factor of the self-diffusion coefficient, $A_{SD}$, is 11.75 cm^2^/s [27]. The vacancy formation entropy $\Delta S_{v}$ is 2.17 k/vacancy [30].

Self-diffusion coefficient ($D_{SD}=A_{SD}\exp\left(-\frac{H_{SD}}{kT}\right)$ is introduced to increase the accuracy in the calculation of $\beta_{v}$, the arrival rates of vacancies to a void per unit area and unit time. For self-diffusion dominated by the monovacancy diffusion mechanism, $D_{SD}$ is given by the following:

(7) $$D_{SD}=f_{v}D_{v}c_{v}^{eq}$$

where $f_{v}$ is the diffusion correlation factor when the sequential jumping directions are correlated. Correlation is always expected when a trace atom diffuses via the vacancy diffusion mechanisms. The most commonly used $f_{v}$ value is 0.72722 for the monovacancy diffusion mechanism in the BCC crystal structure [31,32]. $D_{V}$ is the vacancy diffusivity. $c_{v}^{eq}$ is the vacancy atomic fraction at equilibrium. $D_{v}$ is given by the following:

(8) $$D_{v}=\frac{1}{6}v\lambda^{2}$$

where v is the number of successful jumps per second, and *λ* is the jumping distance. $D_{v}$ is related to $\beta_{v}$ (vacancy flux to a void surface). $\beta_{v}\;$is given by the following:

(9) $$\beta_{v}=\frac{1}{6}v(c_{v}N\lambda )$$

where $c_{v}$ is the vacancy atomic fraction under irradiation, and N is the substrate atomic density (8.482 × 10^22^/cm^3^ for Fe) [33]. The product of $c_{v}$, N, and *λ* gives the number of vacancies in a volume defined by a unit area and the thickness of one jumping distance. The vacancy supersaturation ratio is given by the following:

(10) $$S\equiv \frac{C_{v}}{C_{v}^{eq}}$$

where $C_{v}$ and $C_{v}^{eq}$ are the vacancy concentration and the vacancy equilibrium concentrations, respectively. Combining Equations (7)–(10), one obtains the following:

(11) $$\beta_{v}=\frac{D_{SD}SN}{f_{v}\lambda }$$

Using $D_{SD}$ to obtain $\beta_{v}$ increases the accuracy, since the attempt jumping frequency, which is the pre-exponential factor of v in Equation (9), is difficult to calculate. Another advantage is that *S* appears as a variable in the expression.

## 3. Results

### 3.1. Void Nucleation at Arbitrary T, S, and $\beta_{i}/\beta_{v}$

**Figure 1:**
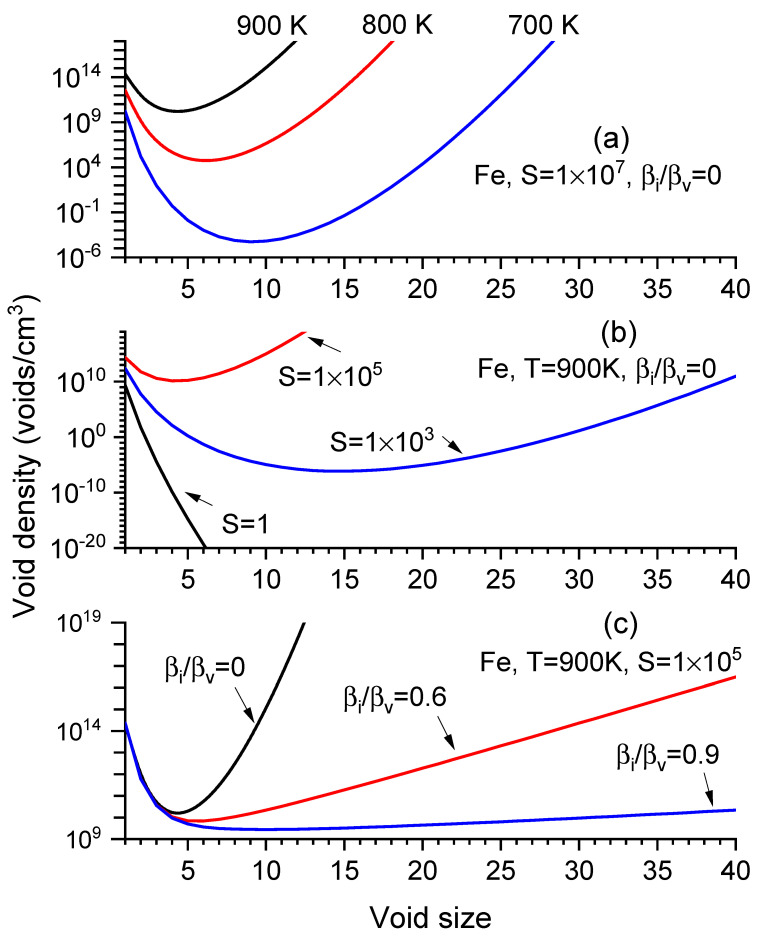
Void density $n\left(x\right)\;$ as a function of void size (number of vacancies contained in a void) under various conditions in α-iron: (**a**) different temperatures, (**b**) different *S* values, and (**c**) different $\beta_{i}/\beta_{v}$ ratios.

As a summary of the results obtained using Equations (4) and (5), Figure 1a–c shows $n\left(x\right)$ distribution changes by varying temperatures (T), vacancy supersaturation ratios S, and the ratios of interstitial-to-vacancy arrival rates to a void ($\beta_{i}/\beta_{v}$). All n(x) curves feature a dip at the critical size, $x_{c}$. With increasing T, the size curves move upwards, exhibiting smaller $x_{c}$ and higher $n(x_{c})$ (Figure 1a). Similarly, with increasing *S* values, the curves shift upward, reducing $x_{c}$ and increasing $n(x_{c})$ (Figure 1b). Increasing $\beta_{i}/\beta_{v}\;$increases $x_{c}$ while reducing void density at large sizes (Figure 1c).

**Figure 2:**
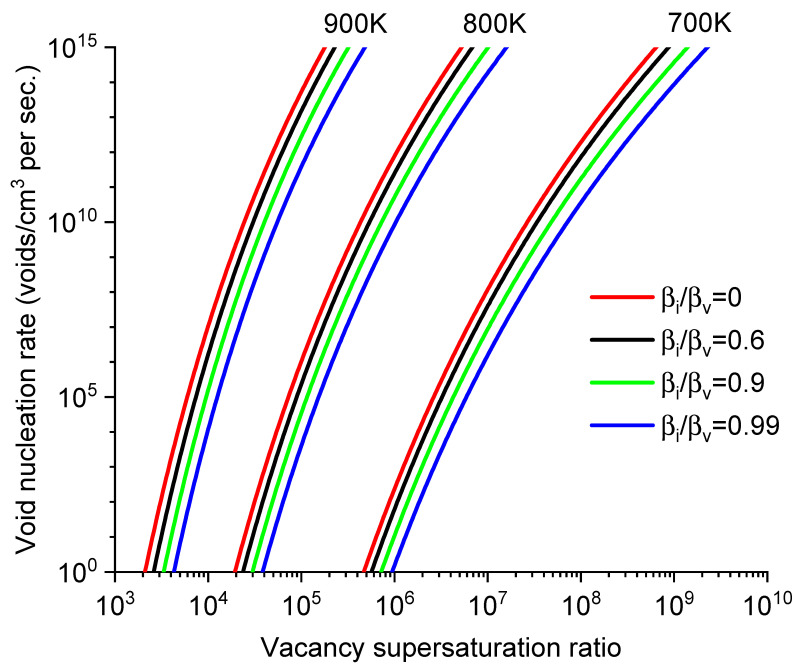
Void nucleation rates as a function of S at different temperatures (700, 800 K, 900 K) and $\beta_{i}/\beta_{v}$ ratios (0, 0.6, 0.9, and 0.99).

Once n(x) is obtained, Equation (2) is used to calculate void nucleation rate *J*. Figure 2 plots the *J* values as a function of *S* for different *T* and $\beta_{i}/\beta_{v}$. The *J* values are very sensitive to all three parameters. For example, at 900 K and $\beta_{i}/\beta_{v}\;$ = 0.99, a change of *S* from 1 × 10^4^ to 1 × 10^5^ leads to an increase in *J* by more than six orders of magnitude. Figure 2 also shows a compensating effect among parameters. For instance, to maintain the same *J* value, the impact of lowering *T* can be counterbalanced by increasing *S*.

### 3.2. Void Nucleation Considering Irradiation for Defect Production and Dislocations as Defect Sinks

After establishing the relationship of n(x) with *T*, S, and $\beta_{i}/\beta_{i}$, the study proceeds to rate theory calculations to establish how these parameters relate to the displacements per atom $K_{0}$ and defect sinks. Two master equations describe the time rate of change of vacancy concentration ($C_{v}$) and interstitial concentration ($C_{i}$), expressed as follows [1]:

(12) $$\frac{\partial C_{v}}{\partial t}=f_{survive}{NK}_{0}+K_{v}^{th}-K_{⊥(v)}\rho_{v}C_{v}-K_{iv}C_{v}C_{i}+\nabla D_{v}\nabla C_{v}$$

(13) $$\frac{\partial C_{i}}{\partial t}=f_{survive}{NK}_{0}+K_{i}^{th}-K_{⊥(i)}\rho_{i}C_{i}-K_{iv}C_{v}C_{i}+\nabla D_{i}\nabla C_{i}$$

where t is time, $f_{survive}$ is the survival fraction of defects after the initial damage creation, N is the atomic density of Fe, $K_{0}$ is the displacements-per-atom (dpa), $K_{v}^{th}$ and $K_{i}^{th}$ are thermal generation rates of vacancies and interstitials, respectively, $K_{⊥(v)}$ and $K_{⊥(i)}$ are sink strength for vacancies and interstitials, respectively, $\rho_{v}$ and $\rho_{i}$ are sink densities for vacancies and interstitials, respectively, $K_{iv}$ is the interstitial-vacancy recombination rate$,D_{v}$ and $D_{i}$ are diffusivities of vacancies and interstitials, respectively.

Under equilibrium conditions, without ion irradiation and diffusion, the above two equations lead to the expression of the thermal generation rates.

(14) $$K_{v}^{\mathrm{th}}=K_{⊥\left(v\right)}\rho_{v}C_{v}^{eq}+K_{\mathrm{iv}}C_{v}^{eq}C_{i}^{eq}$$

(15) $$K_{i}^{\mathrm{th}}=K_{⊥\left(i\right)}\rho_{i}C_{i}^{eq}+K_{\mathrm{iv}}C_{v}^{eq}C_{i}^{eq}$$

Substituting the above two equations into Equations (12) and (13), and assuming a steady state where $\frac{\partial C_{v}}{\partial t}=0$ and $\frac{\partial C_{i}}{\partial t}=0$, the following equation can be easily derived. The equation can also be obtained, without mathematics, but based on the physics that the net point defect fluxes of interstitials and vacancies to defect sinks are equal. Otherwise, a steady state cannot be reached [34].

(16) $$K_{⊥(v)}\rho_{v}\left(C_{v}-C_{v}^{eq}\right)=K_{⊥(i)}\rho_{i}\left(C_{i}-C_{i}^{eq}\right)$$

Combining Equations (12)–(16) for a steady state under irradiation, a quadratic equation is derived for vacancy concentration deviation from its equilibrium $\left(∆C_{v}=C_{v}-C_{v}^{eq}\right)$, expressed as follows:

(17) $$\frac{K_{iv}K_{⊥(v)}\rho_{v}}{K_{⊥(i)}\rho_{i}}{∆C_{v}}^{2}+\left(K_{⊥(v)}\rho_{v}+K_{iv}C_{i}^{eq}+\frac{K_{iv}K_{⊥(v)}\rho_{v}}{K_{⊥(i)}\rho_{i}}C_{v}^{eq}\right)∆C_{v}-f_{survive}NK_{0}=0$$

The solution is as follows:

(18) $$C_{v}=C_{v}^{eq}+\frac{1}{2}a\left(\sqrt{1+\frac{4b}{a^{2}}}-1\right)$$

with

(19) $$a=\frac{K_{⊥(i)}\rho_{i}}{K_{⊥(v)}\rho_{v}}C_{i}^{eq}+C_{v}^{eq}+\frac{K_{⊥(i)}\rho_{i}}{K_{iv}}$$

(20) $$b=\frac{{f_{survive}NK_{0}K}_{⊥(i)}\rho_{i}}{K_{iv}K_{⊥(v)}\rho_{v}}$$

Substituting $C_{v}$ obtained from Equation (18) into Equation (16) obtains $C_{i}$. Then the ratio ${\beta_{i}/\beta }_{v}$ can be obtained using ${\beta_{i}/\beta }_{v}=$($D_{i}C_{i})/{(D}_{v}C_{v})$.

The dislocation sink strength $K_{⊥}\;$is described by the following:

(21) $$K_{⊥(i,v)}=\frac{2\pi D_{\left(i,v\right)}}{ln\left(\frac{1/\sqrt{\pi \rho_{⊥}}}{r_{⊥(i,v)}}\right)}$$

where $\rho_{⊥}$ is dislocation density, and $r_{⊥(i,v)}$ is the defect-trapping radius for trapping interstitials and vacancies. The point defect recombination rate is calculated by the following [1]:

(22) $$K_{iv}=4\pi r_{iv}(D_{i}+D_{v})/\Omega ≅500\Omega D_{i}/a^{2}$$

where Ω is atomic volume of one lattice atom and a is the lattice constant of Fe.

**Figure 3:**
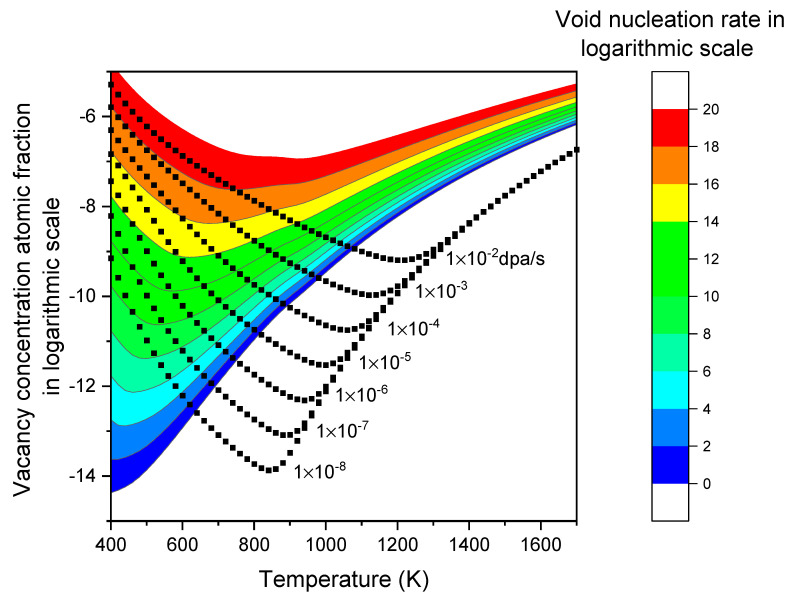
Vacancy concentration (atomic fraction, on a logarithmic scale) at different temperatures for various steady-state void nucleation rates (color contour) and under different dpa rates (square lines) in α-iron. The point of intersection between a square line and a colored contour line gives the nucleation rate at a specific temperature and dpa rate.

Table 2 summarizes the parameters used in the present study. The black square lines in Figure 3 show the calculated vacancy atomic fraction concentration as a function of temperature at various dpa rates. Under a given dpa rate, the vacancy concentration exhibits a V-shaped pattern. At very high temperatures, the concentration follows $C_{v}^{eq}$. As the temperature decreases, $C_{v}$ initially decreases and then begins to rise at a certain temperature point and deviates from $C_{v}^{eq}$. At sufficiently low temperatures, where $C_{v}^{eq}$ and $C_{i}^{eq}$ are negligible, $C_{v}$ becomes proportional to $\sqrt{K_{0}}$. Changing $K_{0}$ by one order of magnitude results in a nearly parallel shift of the logarithmic plot of $C_{v}$ at low temperatures. This shift is obvious when comparing the square lines for $K_{0}$ ranging from 1 × 10^−2^ dpa/s to 1 × 10^−8^ dpa/s. Lowering $K_{0}$ causes the temperature point at which $C_{v}$ deviates from $C_{v}^{eq}$ to shift to lower temperatures accordingly. In Figure 3, the calculations assume a dislocation density of 1 × 10^10^/cm^2^. Changing the dislocation density does not cause a significant shift in the curve, unlike $K_{0}$. Instead, the dislocation density mainly influences the concentration near the turning point. A higher dislocation density results in a lower defect concentration and shifts the turning point to a lower temperature. The effect of dislocation density becomes more evident under lower dpa rate irradiation.

The color bands in Figure 3 represent the contour map of the void nucleation rate as a function of vacancy concentration and temperature. Each color or contour line corresponds to a constant void nucleation rate. It exhibits a V-shaped pattern. For lower void nucleation rates, the minimum point of the V-shaped pattern shifts to lower defect concentrations and temperatures. The V-shaped behavior primarily arises from the change in the critical void size ($x_{c}$). When $x_{c}$ is larger, the void density at that size, $n(x_{c})$, is lower, resulting in lower void nucleation rates. In the high-temperature region (i.e., >900 K), $x_{c}$ decreases with increasing temperature, leading to higher nucleation rates at higher temperatures. The effect of irradiation is less significant in this temperature range because $C_{v}^{eq}$ is already high, and the irradiation-induced *S* changes become insignificant. At extremely high temperatures, S approaches 1. In the low-temperature region (i.e., T < 600 K), the effect of *S* becomes more significant as $C_{v}^{eq}$ is very low. Although lowering the temperature would tend to increase $x_{c}$, the high value of *S* counteracts this and reduces $x_{c}$. The significant changes in S alter the trend, resulting in the V-shaped temperature dependence as shown in Figure 3.

For two points on the contour line with the same nucleation rate, the following observations were made: Taking the example of a nucleation rate of 1 × 10^18^ voids/cm^3^ per second, when the temperature decreases from 700 K to 600 K, the significantly increased vacancy concentration (and subsequently, *S* values) shift the entire n(x) profile upwards, resulting in higher void densities at each size. Conversely, $\beta_{v}$ is reduced when the temperature changes from 700 K to 600 K, due to large changes in vacancy diffusivity. This reduction in $\beta_{v}$ compensates for the increase in void density, thereby maintaining the same nucleation rate.

### 3.3. Void Nucleation Considering Irradiation, Dislocation, and Carbon Incorporation

Carbon is well-known for causing the suppression of void swelling, but no quantitative evaluation has been established to assess its impact, which motivates the present study. The effect was not significant during the short-term ion irradiation of materials in early days but has become significant for prolonged irradiation of current advanced alloys, which are highly swelling-resistant. More recent relevant studies show that carbon is the reason why the width of the void-denuded zone is governed by an activation energy that significantly deviates from the expected vacancy migration energies [12].

C atoms temporarily immobilize vacancies through the formation of V-C_n_ complexes. The complexes are expected to be dissociated later, and the dissociation probability is determined by the V-C binding energies. The binding energies of various C-V complexes were previously calculated using ab initio calculations [17]. The binding energies $E_{b}$ are 0.41 eV for VC, 1.18 eV for VC_2_, and 1.30 eV for VC_3_. The complex concentration, expressed as the atomic fraction concentration, ${\tilde{C}}_{V_{m}C_{n}}$, of various vacancy-carbon complexes $V_{m}C_{n}$ is approximated by the mass-action law [17]:

(23) $${\tilde{C}}_{V_{m}C_{n}}={\left({\tilde{C}}_{v}\right)}^{m}{\left({\tilde{C}}_{C}\right)}^{n}exp(E_{b}/kT)$$

where m is the number of vacancies and n is the number of carbon atoms in a vacancy-carbon complex. $E_{b}$ is the binding energy of the complex.

Under the approximation that (1) the amount of carbon bonded with vacancies in complexes is significantly less than the total carbon dissolved in the system, and (2) the major complexes consist of those containing one vacancy (*m* = 1) and multiple carbons with *n* = 1, 2, and 3, the effective vacancy diffusivity ${D_{v}}^{eff}$ can be calculated by the following:

(24) $${D_{v}}^{eff}=D_{v}\frac{{{\tilde{C}}_{v}}^{free}}{{{\tilde{C}}_{v}}^{free}+{{\tilde{C}}_{v}}^{free}{\tilde{C}}_{C}\exp\left(\frac{E_{b1}}{kT}\right)+{{\tilde{C}}_{v}}^{free}({{\tilde{C}}_{C})}^{2}\exp\left(\frac{E_{b2}}{kT}\right)+{{\tilde{C}}_{v}}^{free}({{\tilde{C}}_{C})}^{3}\exp\left(\frac{E_{b3}}{kT}\right)}\;=\frac{D_{v}}{1+\sum_{n=1}^{3}({{\tilde{C}}_{C})}^{n}\exp\left(\frac{E_{bn}}{kT}\right)}$$

where $D_{v}$ is the intrinsic diffusivity of vacancies, ${{\tilde{C}}_{v}}^{free}$ represents the atomic fraction concentration of free/isolated vacancies, ${\tilde{C}}_{C}$ is the atomic fraction concentration of carbon, and $E_{bn}$ is the binding energy of vacancy-carbon complex $V_{1}C_{n}$ with *n* = 1, 2, and 3.

**Figure 4:**
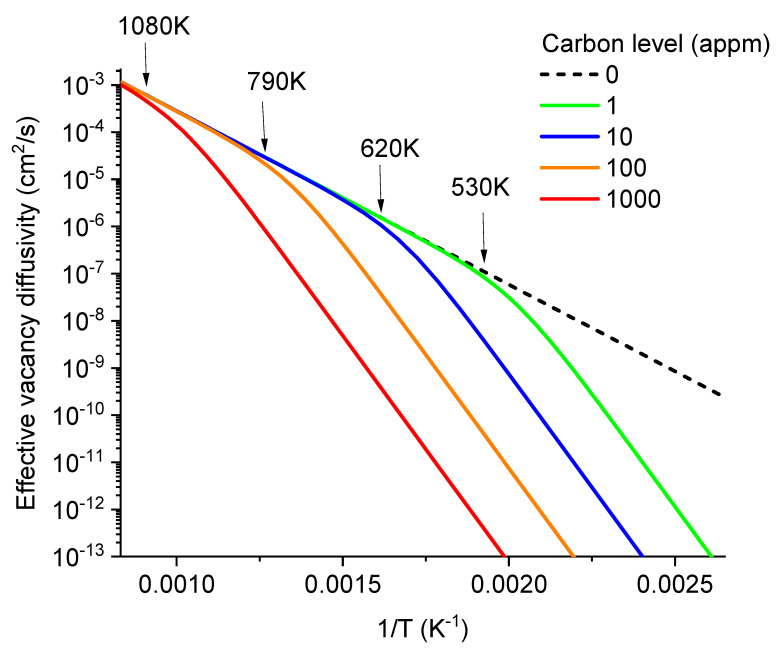
Effective vacancy diffusivity as a function of temperatures and C concentrations in α-iron.

Figure 4 plots the effective vacancy diffusivity as a function of temperature for carbon concentrations at 1, 100, and 10,000 appm levels. The solid line represents C-free Fe, exhibiting a single activation energy (0.73 eV). With the addition of carbon, the effective diffusivity deviates from the solid line at temperatures below a critical temperature. At higher C levels, this deviation occurs at higher temperatures. Notably, in the affected-temperature region, the effective vacancy diffusivity exhibits roughly the same activation energy of 1.91 eV, regardless of the carbon level. The diffusivity curve is parallelly shifted downward with increasing carbon levels. The constant activation energy of 1.91 eV for all C levels can be approximated as the sum of the migration energy (0.73 eV) of free vacancies and the binding energy (1.18 eV) of VC_2_ complexes [17]. The effect of VC_2_ is dominant. The VC complex plays a less significant role due to its much lower E_b_ value, and VC_3_ is also less significant due to its relatively lower concentrations [17]. Figure 4 also marks the temperatures at which the effective vacancy diffusivities begin to deviate from the carbon-free case. As discussed soon, these critical temperature points also play an important role in determining the temperature dependence of void nucleation.

The method of calculating effective vacancy diffusivity in the present study follows the early approach of Fu et al. [17]. The diffusivity calculation in Reference [17] used a vacancy migration energy of 0.67 eV. In the plot of Figure 4, an activation energy of 0.73 eV is used [27], to be consistent with the calculations in the preceding sections.

**Figure 5:**
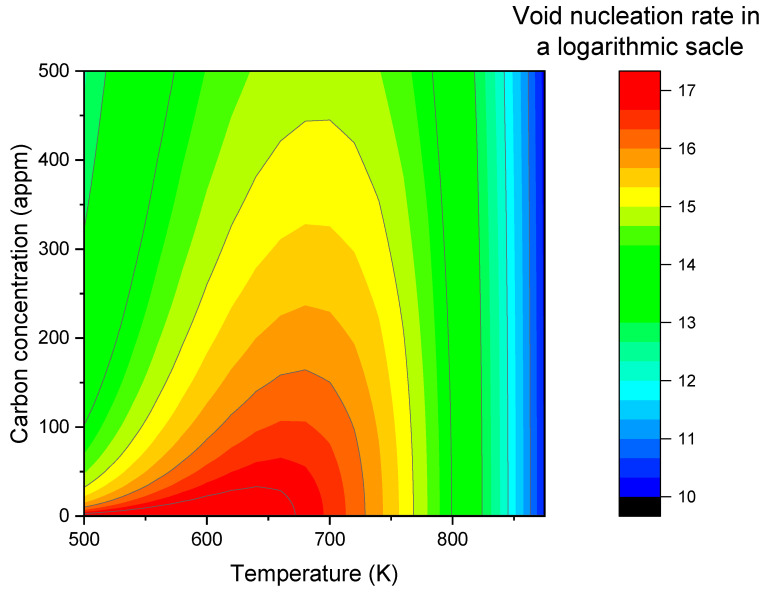
The map of void nucleation rates as a function of C concentrations and temperatures in α-iron.

Substituting ${D_{V}}^{eff}$ into rate theory calculations to determine $S,\;\beta_{v},$ and $\beta_{i}/\beta_{v}$, and then substituting these parameter values into the void nucleation rate calculation, one obtains *J* as a function of both carbon concentration and temperatures. As shown in Figure 5, introducing carbon dramatically changes the contour map. At zero carbon concentration, the nucleation rate remains high at temperature below ~700 K. However, with a small amount of carbon addition, nucleation rates quickly evolve into a temperature-dependent peak. As the carbon concentration increases, the peak height becomes lower, the peak width is narrower, and the peak center slightly shifts to a higher temperature.

**Figure 6:**
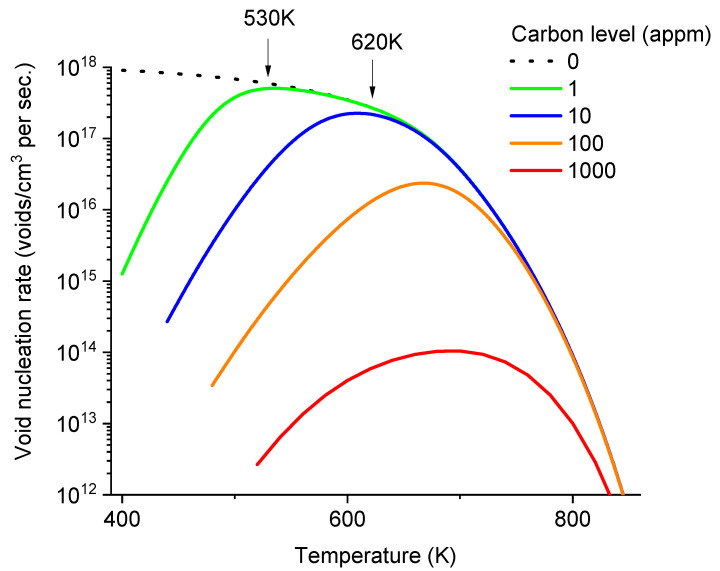
The plot of void nucleation rates as a function of temperature for C concentrations ranging from 0 to 1000 appm in α-iron.

In Figure 6, the void nucleation rates are plotted as a function of temperature while fixing carbon concentrations at distinct levels of 0, 1, 10, 100, and 1000 appm. At zero carbon concentration (dot line), *J* remains above 3 × 10^17^/cm^3^ per second when temperature is at 600 K and below. Even with carbon levels as low as 1 appm, void nucleation begins to show a peak. The nucleation rates start to drop at temperatures ~530 K and below. At a higher carbon level of 10 appm, the nucleation allowable temperature window is narrower, and the temperature of the maximum void nucleation rate increases to about 600 K. Additionally, at this carbon level, the peak nucleation rate is noticeably lower than in the carbon-free case. As shown by two arrows (one at 530 K for 1 appm carbon and the other at 620 K for 10 appm carbon), the temperatures at which void nucleation begins to deviate from the carbon-free case correspond to the temperatures at which effective diffusivities of vacancies begin to deviate from the carbon-free case (as marked in Figure 4). At a higher carbon level of 100 appm, the peak shifts to about 670 K, and the peak height is about 25% of that in the carbon-free case at the same temperature. At the highest carbon level of 1000 appm, the nucleation peak is reduced by more than two orders of magnitude compared to the carbon-free curve at the same temperature.

One way to validate the carbon effect on effective vacancy diffusivity is to measure the width of the void-denuded zone, denoted by ∆x. According to the analytical solution of rate theory equations, $∆x\propto {{(D}_{V}/K)}^{1/4}$ [38]. Recent studies on Fe irradiated by self-ions at various temperatures, beam energies, and dpa rates have measured an effective vacancy activity energy of 1.65 eV for single crystal Fe containing a carbon background concentration of about 100 appm [12]. The experimentally extracted value of 1.65 eV aligns with the 1.91 eV predicted from Figure 4 and is significantly larger than the 0.73 eV predicted for carbon-free cases.

Validation of void nucleation in carbon-free Fe is indeed very challenging due to various factors, including the difficulty of manufacturing and irradiation testing. Carbon contamination has been an issue in prolonged accelerator-based ion irradiation testing, and void disappearance has been frequently reported [39,40,41]. Carbon contamination is not expected in reactor irradiation testing. However, parameters such as dpa rate, temperature, and neutron flux are all interlinked and coupled. Fixing other parameters and allowing temperature to be the single variable proves to be challenging in reactor experiments. The prediction of a non-zero void nucleation rate in carbon-free Fe at low temperatures is a subject that will be of interest for future studies, especially if low-temperature neutron irradiation can be achieved.

Testing low-temperature void nucleation is challenging in accelerator-based irradiation, with complexities extending beyond beam contamination. In the case of accelerator-based ion irradiation, heavy ions suffer from the injected interstitial effect [42,43,44], which exhibits strong temperature dependence [44]. The swelling suppression by the injected interstitials becomes more significant at lower temperatures [44], leading to a suppression behavior similar to that of carbon. Proton irradiation can minimize the injected interstitial effects to a large extent. However, proton irradiation can introduce local beam heating, making it difficult to test temperatures lower than 600 K. Commercial tandem accelerators used in university laboratories typically can obtain proton beam currents at a few microamperes. For advanced ion sources, beam currents up to tens of microamperes can be achieved. With typical proton beam energy at a few MeV, the amount of energy deposition is significant. Using a weak proton beam is not realistically helpful since a strong beam is needed to achieve a reasonable damage level.

The findings presented in this study can provide insights into the significant data scattering observed in additively manufactured (AM) steels. Previous reports have indicated that AM steels (316 L) exhibit reduced void swelling compared to wrought materials [45,46]. However, it is noteworthy that void swelling behavior can vary significantly among AM steels manufactured by different groups. One likely cause of such variation is the difficulty in controlling impurities during the additive manufacturing process. These impurities include elements such as carbon, oxygen, and nitrogen, as well as ambient gases used during the printing process. The presence and concentration of these impurities can have a considerable impact on the void-swelling behavior of AM steels.

The high sensitivity of void swelling to carbon levels suggests that it is possible to alloy steels with a small amount of carbon. This can be done at a level that does not significantly alter the optimized mechanical properties but is sufficient to improve the resistance to void swelling. Indeed, nitrogen is another element that can have a similar effect on void swelling in steels. For instance, nitrogen-doped full-ferritic HT-9 exhibits much less swelling compared to conventional HT-9 [47].

The present study is useful in the development of modeling methodologies. As explained in the introduction, the effect of carbon contamination is well known but has never been quantitatively evaluated. This study provides a method to analyze the carbon effect on void nucleation. In void swelling, nucleation during the incubation period is the most significant stage for determining the radiation tolerance of alloys. The proposed method includes the sequential steps of (a) calculating the effective diffusivity of vacancies in the presence of carbon at different levels; (b) calculating defect fluxes and defect supersaturation once effective diffusivity is known from step (a) and the density of pre-existing dislocations is known; and (c) calculating void nucleation rates. This approach can be applied to other metals. The required defect kinetics and carbon-vacancy complex bonding energy can be calculated from atomic-scale simulations.

## 4. Conclusions

The nucleation of voids in alpha-iron under particle irradiation and the influence of carbon were investigated through a combination of rate theory and nucleation theory calculations. The major finding is that the steady rates of void nucleation exhibit high sensitivity to carbon. Even a seemingly negligible carbon concentration, as low as a few appm, can dramatically reduce the nucleation rates and narrow the temperature window for nucleation. Such high sensitivity to carbon impurities comes from the effectiveness of carbon in trapping vacancies and reducing their effective diffusivity, and the importance of vacancy diffusivity in determining void nucleation rates. This study highlights the challenges in certifying and qualifying nuclear materials for use in reactor environments. It emphasizes the necessity for precise control of impurity levels during material processing and stringent requirements for irradiation testing, which are susceptible to contamination. Furthermore, the study provides an explanation for the significant data scattering observed in void swelling behaviors as reported in the literature.

## Figures and Tables

**Table 1:** Parameters used in the present study for calculating void nucleation rates.

Parameters	Values	References
Vacancy formation enthalpy $H_{v}^{f}$ (eV)	2.59	[25]
Vacancy migration enthalpy $H_{v}^{m}$ (eV)	0.73	[27]
Pre-exponential factor of self-diffusion coefficient, $A_{SD}$ (cm^2^/s)	11.75	[27]
Activation energy of self-diffusion coefficient, $H_{SD}$ (eV)	3.3	[27]
Vacancy formation entropy $\Delta S_{v}$ (k/vacancy)	2.17	[30]

**Table 2:** Parameters used in the present study for rate theory calculations.

Parameters	Values	References
Vacancy migration enthalpy $H_{v}^{m}$ (eV)	0.73	[27]
Vacancy diffusivity prefactor $D_{0_{v}}$ (${cm}^{2}/s)$	1.34	
Interstitial migration enthalpy $H_{i}^{m}$ (eV)	0.34	[35]
Interstitial diffusivity prefactor $D_{0_{i}}$ (${cm}^{2}/s$)	$2.09\times 10^{-3}$	[36]
Dislocation trapping radius for vacancies $r_{⊥(v)}$ (nm)	1.2	[37]
Dislocation trapping radius for interstitials $r_{⊥(i)}$ (nm)	3.6	[37]
Dislocation density $\rho_{⊥}$ (${cm}^{-2}$)	$10^{10}$	
Survival fraction of defects after damage cascade creation $f_{survive}$	1	

## Data Availability

The datasets analyzed during the current study are available from the corresponding author upon reasonable request.

## Appendix A

The following mathematical derivation is provided based on the reviewers’ suggestions. Readers can find additional details in reference [21].

$n^{0}(x)$ is a special size distribution in which voids and vacancies reach equilibrium. This distribution also has zero net flux of growth when considering vacancy-driven growth and decay of voids. Hence, it satisfies the following condition:

(A1) $$\beta_{v}s\left(x\right)n^{0}\left(x\right)-\gamma_{v}\left(x+1\right)s\left(x+1\right)n^{0}\left(x+1\right)=0$$

In order for $n^{0}(x)$ to reach equilibrium with interstitials as well, a specific interstitial concentration is needed. This concentration is denoted as $\beta_{i}^{0},\;$satisfying the following condition:

(A2) $$-\beta_{i}^{0}s\left(x+1\right)n^{0}\left(x+1\right)+\gamma_{i}\left(x\right)s\left(x\right)n^{0}\left(x\right)=0$$

The above two equations provide expressions for $\gamma_{v}$ and $\gamma_{i}$. The next step is substituting the expressions of $\gamma_{v}$ and $\gamma_{i}$ back into the following equation describing a zero net flux J, which is obtained by balancing the vacancy-driven net flux and the interstitial-driven net flux, as follows:

(A3) $$\beta_{v}s\left(x\right)n\left(x\right)-\gamma_{v}\left(x+1\right)s\left(x+1\right)n\left(x+1\right)-\beta_{i}s\left(x+1\right)n\left(x+1\right)+\gamma_{i}\left(x\right)s\left(x\right)n\left(x\right)=0$$

The above steps obtain a recursive equation for$\;n\left(x\right)$, satisfying the following:

(A4) $$\frac{n(x+1)}{n(x)}=P_{x}\frac{n^{0}(x+1)}{n^{0}(x)}$$

with

(A5) $$P_{x}\equiv \frac{\beta_{v}/m^{0}(x,x+1)+\beta_{i}^{0}}{\beta_{v}/m^{0}(x,x+1)+\beta_{i}}$$

and

(A6) $$m^{0}(x,x+1)\equiv \frac{s(x+1)n^{0}(x+1)}{s(x)n^{0}(x)}$$

Equation (A4) has the well-known solution:

(A7) $$n\left(x\right)=n^{0}(x)\prod_{j=1}^{x-1}P_{j}$$

Substituting Equation (A3) back into Equation (1), and using Equation (A2) to eliminate $\gamma_{i}\left(x\right)$, one obtains the following:

(A8) $$\frac{J\left(x,t\right)}{\left(\beta_{v}+\beta_{i}^{0}m^{0}(x,x+1)\right)s\left(x\right)n(x)}=\left(\frac{f(x,t)}{n(x)}-\frac{f(x+1,t)}{n(x+1)}\right)$$

Summing size n from n=1 to n≫1, and under the following boundary conditions:

$$\frac{f\left(1,t\right)}{n\left(1\right)}=1\;\underset{x\to \infty }{\lim}\frac{f\left(x,t\right)}{n\left(x\right)}=0$$

one obtains

(A9) $$\sum_{x=1}^{x\to \infty }\frac{J\left(x,t\right)}{\left(\beta_{v}+\beta_{i}^{0}m^{0}(x,x+1)\right)s\left(x\right)n(x)}=1$$

Under a steady-state growth with a constant net flux, J is moved outside of the summation, resulting in the following expression:

(A10) $$J={\left[\sum_{x=1}^{x\to \infty }\frac{1}{\left(\beta_{v}+\beta_{i}^{0}m^{0}(x,x+1)\right)s\left(x\right)n(x)}\right]}^{-1}$$

Note that $\beta_{i}^{0}$ is proportional to the interstitial concentration, which reaches equilibrium with voids formed under a specific vacancy supersaturation. $\beta_{i}^{0}$ is very small, it can be disregarded as an approximation, resulting in the following:

(A11) $$J={\left[\sum_{x=1}^{x\to \infty }\frac{1}{\beta_{v}s\left(x\right)n(x)}\right]}^{-1}$$

## References

[1] Was G.S. (2017). Fundamentals of Radiation Materials Science: Metals and Alloys.

[2] Busby J.T., Was G.S., Kenik E.A. (2002). Isolating the effect of radiation-induced segregation in irradiation-assisted stress corrosion cracking of austenitic stainless steels. J. Nucl. Mater..

[3] Cawthorne C., Fulton E.J. (1967). Voids in irradiated stainless steel. Nature.

[4] Khvostov G. (2018). Modeling of central void formation in LWR fuel pellets due to high-temperature restructuring. Nucl. Eng. Technol..

[5] Huang F.H., Mills W.J. (1991). Delayed hydride cracking behavior for ZIRCALOY-2 tubing. J. Mater. Sci..

[6] Edsinger K., Davies J., Adamson R., Sabol G., Moan G. (2000). Degraded Fuel Cladding Fractography and Fracture Behavior. Zirconium in the Nuclear Industry: Twelfth International Symposium.

[7] Chopra O.K., Rao A.S. (2011). A review of irradiation effects on LWR core internal materials—Neutron embrittlement. J. Nucl. Mater..

[8] Murty K.L., Gollapudi S., Ramaswamy K., Mathew M.D., Charit I., Murty K.L. (2013). Creep deformation of materials in light water reactors (LWRs). Materials Ageing and Degradation in Light Water Reactors.

[9] Zinkle S.J., Ghoniem N.M. (2000). Operating temperature windows for fusion reactor structural materials. Fusion Eng. Des..

[10] Barabash V., Kalinin G., Kawamura H., Mazul I., Fabritsiev S. (2000). Materials challenges for ITER—Current status and future activities. J. Nucl. Mater..

[11] Garner F.A., Konings R.J.M., Stoller R.E. (2020). Radiation-Induced Damage in Austenitic Structural Steels Used in Nuclear Reactors. Comprehensive Nuclear Materials.

[12] Li Y., Hu Z., French A., Cooper K., Garner F.A., Shao L. (2023). Effect of the free surface on near-surface void swelling in self-ion irradiated single crystal pure iron considering the carbon effect. J. Nucl. Mater..

[13] Bhadeshia H.K.D.H. (2004). Carbon-carbon interaction in iron. J. Mater. Sci..

[14] Domain C., Becquart C.S., Fort J. (2004). Ab initio study of foreign interstitial atom (C N) interactions with intrinsic point defects in α-Fe. Phys. Rev. B.

[15] Jourdan T., Fu C.C., Joly L., Bocquet J.L., Caturla M.J., Willaime F. (2011). Direct simulation of resistivity recovery experiments in carbon-doped α-iron. Phys. Scr..

[16] Hashimoto N., Sakuraya S., Tanimoto J., Ohnuki S. (2014). Effect of impurities on vacancy migration energy in Fe-based alloys. J. Nucl. Mater..

[17] Fu C.C., Meslin E., Barbu A., Willaime F., Oison V. (2008). Effect of C on Vacancy Migration in α-Iron. Solid State Phenom..

[18] Kim H., Gigax J.G., El-Atwani O., Chancey M.R., Baldwin J.K., Wang Y., Maloy S.A. (2021). Comparison of void swelling of ferritic-martensitic and ferritic HT9 alloys after high-dose self-ion irradiation. Mater. Charact..

[19] Was G.S., Taller Z., Jiao Z., Monterrosa A.M., Woodley D., Jennings D., Kubley T., Naab F., Toader O., Uberseder E. (2017). Resolution of the carbon contamination problem in ion irradiation experiments. Nucl. Instrum. Methods Phys. Res. B.

[20] Shao L., Gigax J., Chen D., Kim H., Garner F.A., Wang J., Toloczko M.B. (2017). Standardization of accelerator irradiation procedures for simulation of neutron induced damage in reactor structural materials. Nucl. Instrum. Methods Phys. Res. B.

[21] Katz J.L., Wiedersich H. (1971). Nucleation of voids in materials supersaturated with vacancies and interstitials. J. Chem. Phys..

[22] Russell K.C. (1971). Nucleation of voids in irradiated metals. Acta Metall..

[23] Shao L. (2024). Homogeneous void nucleation in the presence of supersaturated vacancies and interstitials. J. Materiomics.

[24] Hou J., You Y.W., Kong X.S., Song J., Liu C.S. (2021). Accurate prediction of vacancy cluster structures and energetics in bcc transition metals. Acta Mater..

[25] Soneda N., Diaz de la Rubia T. (1998). Defect production, annealing kinetics and damage evolution in α-Fe: An atomic-scale computer simulation. Philos. Mag. A.

[26] Li Y., French A., Hu Z., Gabriel A., Hawkins L.R., Garner F.A., Shao L. (2023). A quantitative method to determine the region not influenced by injected interstitial and surface effects during void swelling in ion-irradiated metals. J. Nucl. Mater..

[27] Shang S.L., Zhou B.C., Wang W.Y., Ross A.J., Liu X.L., Hu Y.J., Fang H.Z., Wang Y., Liu Z.K. (2016). A comprehensive first-principles study of pure elements: Vacancy formation and migration energies and self-diffusion coefficients. Acta Mater..

[28] Iijima Y., Kimura K., Hirano K. (1988). Self-diffusion and isotope effect in α-iron. Acta Metall..

[29] Lübbehusen M., Mehrer H. (1990). Self-diffusion in α-iron: The influence of dislocations and the effect of the magnetic phase transition. Acta Metall. Mater..

[30] Burton J.J. (1972). Vacancy-formation entropy in cubic metals. Phys. Rev. B.

[31] Compaan K.C., Haven Y. (1956). Correlation factors for diffusion in solids. Trans. Faraday Soc..

[32] Peterson N.L. (1978). Self-diffusion in pure metals. J. Nucl. Mater..

[33] Callister W.D., Rethwisch D.G. (2014). Materials Science and Engineering: An Introduction.

[34] Wiedersich H. (1972). On the theory of void formation during irradiation. Radiat. Eff..

[35] Fu C.C., Willaime F., Ordejón P. (2004). Stability and mobility of mono- and di-interstitials in α-Fe. Phys. Rev. Lett..

[36] Soneda N., Diaz de La Rubia T. (2001). Migration kinetics of the self-interstitial atom and its clusters in bcc Fe. Philos. Mag. A.

[37] Shastry V., Diaz de la Rubia T. (1999). The Interaction Between Point Defects and Edge Dislocation in BCC Iron. ASME J. Eng. Mater. Technol..

[38] Lam N.Q., Rothman S.J., Sizmann R. (1974). Steady-state point-defect diffusion profiles in solids during irradiation. Radiat. Eff..

[39] Williams T.M. (1980). The effect of soluble carbon on void swelling and low dose dislocation structures in type 316 austenitic stainless steel irradiated with 46.5 MeV Ni^6+^ ions. J. Nucl. Mat..

[40] Wang J., Toloczko M.B., Kruska K., Schreiber D.K., Edwards D.J., Zhu Z., Zhang J. (2017). Carbon contamination during ion irradiation—Accurate detection and characterization of its effect on microstructure of ferritic/martensitic steels. Sci. Rep..

[41] Gigax J.G., Kim H., Aydogan E., Garner F.A., Maloy S., Shao L. (2017). Beam-contamination-induced compositional alteration and its neutron-atypical consequences in ion simulation of neutron-induced void swelling. Mater. Res. Lett..

[42] Shao L., Wei C.C., Gigax J., Aitkaliyeva A., Chen D., Sencer B.H., Garner F.A. (2014). Effect of defect imbalance on void swelling distributions produced in pure iron irradiated with 3.5 MeV self-ions. J. Nucl. Mater..

[43] Short M.P., Gaston D.R., Jin M., Shao L., Garner F.A. (2016). Modeling injected interstitial effect on void swelling in self-ion irradiation experiments. J. Nucl. Mater..

[44] Plumton D.L., Wolfer W.G. (1984). Suppression of void nucleation in injected interstitials during heavy ion bombardment. J. Nucl. Mater..

[45] Song M., Wang M., Lou X., Rebak R.B., Was G.S. (2019). Radiation damage and irradiation-assisted stress corrosion cracking of additively manufactured 316L stainless steels. J. Nucl. Mater..

[46] Shiau C.H., McMurtrey M.D., O’Brien R.C., Jerred N.D., Scott R.D., Lu J., Zhang X., Wang Y., Shao L., Sun C. (2021). Deformation behavior and irradiation tolerance of 316L stainless steel fabricated by direct energy deposition. Mater. Des..

[47] Kim H., Gigax J.G., Rietema C.J., El Atwani O., Chancey M.R., Baldwin J.K., Wang Y., Maloy S.A. (2022). Void swelling of conventional and composition engineered HT9 alloys after high-dose self-ion irradiation. J. Nucl. Mater..

